# Editorial: Neurological and neuropsychiatric disorders affecting military personnel and veterans, volume II

**DOI:** 10.3389/fneur.2026.1923368

**Published:** 2026-08-12

**Authors:** Lisa C. Krishnamurthy, Venkatagiri Krishnamurthy, Mary Jo Pugh, William C. Walker, Chen Lin

**Affiliations:** 1 Joseph Maxwell Cleland Atlanta Veteran's Affairs (VA) Medical Center, Decatur, GA, United States; 2 Department of Radiology and Imaging Sciences, Emory University, Atlanta, GA, United States; 3 Department of Rehabilitation Medicine, Emory University, Atlanta, GA, United States; 4 Department of Biomedical Engineering, Georgia Tech and Emory University, Atlanta, GA, United States; 5 Division of Geriatrics and Gerontology, Department of Medicine, Emory University, Atlanta, GA, United States; 6 Department of Neurology, Emory University, Atlanta, GA, United States; 7 VA Salt Lake City Health Care System, Informatics, Decision-Enhancement and Analytic Sciences Center, Salt Lake City, UT, United States; 8 Division of Epidemiology, University of Utah School of Medicine, Salt Lake City, UT, United States; 9 Department of Physical Medicine and Rehabilitation, Virginia Commonwealth University, Richmond, VA, United States; 10 Department of Neurology, Louisiana State University Health, Shreveport, LA, United States

**Keywords:** chronic pain, cognitive impairment, military personnel, polytrauma, post-traumatic stress disorder, rehabilitation, traumatic brain injury, veterans

Military personnel and Veterans experience neurological and neuropsychiatric health challenges that are shaped by the distinctive demands, exposures, and consequences of military service. These challenges include traumatic brain injury (TBI) of all severity, including concussion, post-traumatic stress disorder (PTSD), chronic pain, stroke, epilepsy, sensory impairment, cognitive dysfunction, depression, and dementia-related risk (1–3). Yet the central lesson from this Research Topic is that these conditions rarely occur in isolation. They often emerge as interacting clinical syndromes that unfold across time, intersect with physical disability and psychological distress, and are shaped by barriers to recognition, access to care, stigma, family context, and the structure of military and Veteran health systems (4–6).

The first theme of this volume is that military-related neurological injury may have consequences that extend well beyond the period of active service. Concussion and more severe TBI are often framed around acute diagnosis, return to duty, or short-term recovery, but the long-term consequences may become increasingly visible as military cohorts age. In this context, the association between service-related TBI and later diagnoses of memory loss, mild cognitive impairment, and dementia-related outcomes underscores the need to view brain injury as a lifespan health issue (Belding et al.). These findings point toward the importance of long-term surveillance, early cognitive assessment, and rehabilitation planning for Veterans whose neurological risk may evolve over decades rather than months.

A second theme is that neurological and neuropsychiatric disorders in military and Veteran populations require models that move beyond single diagnoses. Several contributions in this volume show that clinical risk is shaped not only by whether a person has TBI, epilepsy, PTSD, chronic pain, or depression, but also by how these conditions interact, when they occur, and how they organize into cognitive, behavioral, and functional phenotypes. For example, the temporal sequence of TBI and epilepsy appears to matter, with different patterns of cognitive and behavioral vulnerability emerging depending on whether TBI preceded epilepsy or followed it (Panahi et al.). Similarly, the polytrauma clinical triad of PTSD, chronic pain syndromes, and TBI is best understood not as three separate problems occurring side by side, but as an interacting, polysymptomatic condition that can constrain recovery (Manhapra et al.). Together, these articles argue for phenotype-informed and multimorbidity-informed care models that better reflect the complexity of real-world Veteran health.

A third theme is that better care begins with better recognition. Some symptoms are missed because they are under-recognized; others are mislabeled because they resemble psychiatric, cognitive, or neurological disorders with overlapping features. Charles Bonnet Syndrome in visually impaired Veterans is one example: visual hallucinations may be distressing and stigmatized, yet they can be misattributed to dementia, psychosis, PTSD, or cognitive decline (McCready et al.). The co-production of accessible educational tools for Veterans, caregivers, and clinicians illustrates how improved recognition can reduce fear, support disclosure, and guide appropriate care. In the context of concussion rehabilitation, the challenge is slightly different but equally important: broad complaints such as “memory problems” or “attention problems” must be translated into specific, everyday functional targets. Activity-focused tools such as the Common Cognitive Complaints after Concussion questionnaire help bridge this gap by linking patient-reported problems to individualized rehabilitation goals (McDonald et al.). Across these examples, recognition is not merely diagnostic; it is the first step toward meaningful intervention.

This volume also expands the concept of military-related health burden beyond the individual service member or Veteran. Military service and wartime exposure reverberate through families, shaping psychological distress, somatic symptoms, sleep, pain, and healthcare utilization among those who support service members. The gendered patterns observed among parents of actively serving soldiers highlight that distress is not only experienced differently across individuals, but also organized and expressed differently across social and caregiving roles. For mothers, distress may be more tightly integrated across psychological, somatic, and healthcare-use domains; for fathers, distress may be more compartmentalized and therefore less visible to care systems (Hamama-Raz and Hamama). This work broadens the field's focus from individual pathology to family- and systems-level consequences of military service.

Finally, the Research Topic points toward a future of mechanism-informed, multimodal, and non-pharmacologic intervention. Several articles move beyond documenting burden to testing or conceptualizing interventions that engage brain, body, behavior, and function. Accelerated intermittent theta burst stimulation for depressive symptoms after concussion reflects a precision neuromodulation approach, particularly when guided by individualized functional neuroimaging (Oberman et al.). Pairing neurostimulation with exercise for persistent post-stroke headache similarly reflects a mechanistic strategy aimed at modulating pain networks while engaging activity-dependent plasticity (Lin et al.). Exercise-based approaches for PTSD further emphasize the potential for interventions that affect psychological wellbeing, neuroendocrine regulation, inflammation, sleep, and brain health (Zhao et al.). Across these contributions, the direction is clear: treatment development for military and Veteran populations is increasingly focused on interventions that are biologically plausible, functionally meaningful, and adaptable to complex clinical presentations.

At the same time, this volume makes clear that innovation must be paired with implementation. Promising interventions will have limited impact if they are inaccessible to the Veterans and service members who need them most. Travel burden, transportation barriers, stigma, reluctance to seek care, fragmented services, and the practical demands of military or post-military life can all limit participation (McCready et al.; Hamama-Raz and Hamama; Lin et al.). Future work must therefore consider not only whether an intervention works, but also whether it can be delivered in ways that are acceptable, scalable, and responsive to the lived realities of military-connected individuals and families.

Taken together, the contributions to this Research Topic show that neurological and neuropsychiatric disorders affecting military personnel and Veterans are long-term, heterogeneous, and frequently multisystem conditions. They require careful attention to injury history, comorbidity, symptom phenotype, functional priorities, family context, access barriers, and mechanisms of recovery. The field is advancing from documenting burden toward building tools, models, and interventions that support more precise, accessible, and recovery-oriented care. Continued progress will require longitudinal studies, rigorous clinical trials, biomarker-informed and patient-centered phenotyping, and implementation strategies that meet military-connected individuals and families where they are. This second volume reinforces the need for integrated approaches that recognize the full complexity of military and Veteran health while remaining focused on the ultimate goal: improving function, quality of life, and long-term recovery.
